# A workflow for viewing biomedical computational fluid dynamics results and corresponding data within virtual and augmented reality environments

**DOI:** 10.3389/fmedt.2023.1096289

**Published:** 2023-02-23

**Authors:** John Venn, Christopher E. Larkee, Guilherme J. M. Garcia, Vitaliy L. Rayz, John F. LaDisa

**Affiliations:** ^1^ Department of Biomedical Engineering, Marquette University and Medical College of Wisconsin, Milwaukee, WI, United States; ^2^ Opus College of Engineering, Marquette University, Milwaukee, WI, United States; ^3^ Weldon School of Biomedical Engineering, Purdue University, West Lafayette, IN, United States; ^4^ Department of Pediatrics - Division of Cardiology, Herma Heart Institute, Children’s Wisconsin and the Medical College of Wisconsin, Milwaukee, WI, United States

**Keywords:** simulation - computers, Extended reality (XR), hemodynamics, fluid structure interaction and CFD, patient-specific 3D model

## Abstract

Researchers conducting computational fluid dynamics (CFD) modeling can spend weeks obtaining imaging data, determining boundary conditions, running simulations and post-processing files. However, results are typically viewed on a 2D display and often at one point in time thus reducing the dynamic and inherently three-dimensional data to a static image. Results from different pathologic states or cases are rarely compared in real-time, and supplementary data are seldom included. Therefore, only a fraction of CFD results are typically studied in detail, and associations between mechanical stimuli and biological response may be overlooked. Virtual and augmented reality facilitate stereoscopic viewing that may foster extraction of more information from CFD results by taking advantage of improved depth cues, as well as custom content development and interactivity, all within an immersive approach. Our objective was to develop a straightforward, semi-automated workflow for enhanced viewing of CFD results and associated data in an immersive virtual environment (IVE). The workflow supports common CFD software and has been successfully completed by novice users in about an hour, demonstrating its ease of use. Moreover, its utility is demonstrated across clinical research areas and IVE platforms spanning a range of cost and development considerations. We are optimistic that this advancement, which decreases and simplifies the steps to facilitate more widespread use of immersive CFD viewing, will foster more efficient collaboration between engineers and clinicians. Initial clinical feedback is presented, and instructional videos, manuals, templates and sample data are provided online to facilitate adoption by the community.

## 1. Introduction

Predicting the impact of fluid flow in biomedical applications is time consuming and mathematically complex without computational tools. Computational fluid dynamics (CFD) is a method of simulating fluid passing through or around an object, in the case of arteries and vessels for the current applications, by replacing partial differential equations governing the flow with algebraic equations that can be solved numerically using digital computers. Biomedical CFD therefore yields spatial and temporal patterns of fluid flow that are difficult to measure experimentally, costly to obtain routinely, and potentially intractable due to non-existent clinical methods or the emergent state of a given pathologic situation (1, 2).

CFD researchers with a biomedical focus can spend weeks or months creating realistic geometric models, determining appropriate boundary conditions, running numerical simulations, and post-processing results (3). The results are typically viewed on a 2D display at one point in time, or by slowly progressing through sequential time steps without the aid of supplementary data. Issues with this traditional approach are that only a fraction of the available CFD results are being studied in detail, and potentially inefficiently. For example, a common approach in one co-authors lab is to run simulations for 3–8 cardiac cycles with ∼2,500 to >25,000 time steps per cycle (4), depending on CFL number. Historically, such simulations have taken days to months of physical time to complete. Results that are generated include scalar and vector quantities mentioned below that are then used to calculate additional indices believed to be of clinical and/or biological importance (e.g., wall shear stress) at each element for vessel geometries discretized into tetrahedral meshes of up to 8 million elements (5). Rather than viewing results at each time step and element location, 50 time-steps have traditionally been extracted from simulation results for corresponding videos (∼50 frames-per-second). Results were also traditionally used to construct temporal waveforms at inlet or outlet services, and indices of interest were viewed side-by-side at specific time points (3). Another large limitation with traditional approaches to CFD is that resulting associations from related data can go unnoticed since they are not often viewed in concert with other available data. This is an important point because biomedical CFD simulations are often conducted to better appreciate the relationship between mechanical stimuli and biological response, which can be complex and challenging to visualize without access to complementary information easily viewed in the local proximity of a vessel. Immersive visualization through tools like virtual reality (VR) and augmented reality (AR) can, in theory, alleviate many of these issues by taking advantage of improved depth cues, custom interactivity and development to simultaneously view many sources of related data using an immersive approach that can create a sense of presence within the data for a CFD researcher (6).

The first known attempt to view biomedical CFD results immersively was documented in 2000 when a coronary artery graft was viewed in an IVE to better uncover approaches aimed at reducing failure rates (7). Feedback was mostly positive, but it was noted that “significant time” was put toward reformatting the simulation results before they could be viewed immersively. A common issue presenting when preparing CFD results for immersive viewing is that there is a paucity of approaches that store 3D geometry and vertex magnitude in a format that is natively compatible with related software. A 2010 study aided the process of viewing CFD results in a IVE by developing a semi-automatic workflow that used MATLAB (The MathWorks; Natick, MA) as an intermediary tool to convert select CFD results into files that were compatible with a specific software platform used within an IVE (6). The workflow imitated pulsatile flow in arteries by using time-varying velocity vectors. Velocity vectors for each time step had the same coordinates, but their colors and lengths changed depending on the speed at each location for a particular time step. Blood flow within the featured arteries therefore appeared as though it was pulsating when time steps were displayed rapidly in succession. The tools developed for this application lacked accessibility given the software used to display the results in VR were specific to the IVE within the facility where it was used. Consequently, more advanced functionality was difficult to test and implement. More specifically, preparation of the CFD results featured in the IVE was conducted on in-house computer software packages and using scripts where only the individuals who built the models, ran the simulations, and prepared the visualizations could advance resulting data to the point of use within the specified IVE. In 2015, a study outside of biomedical research viewed CFD results for urban planning using Unity (Unity Technologies; San Francisco, CA) (8). Unity is a multipurpose game engine that is compatible with many immersive systems from head mounted displays (HMD) to large-scale IVEs. Using an approachable and well-documented toolset like Unity may allow more users and labs to create and view their simulation results immersively, potentially alleviating many of the limitations experienced previously with CFD results visualization.

The objective of the current work was to develop a rapid, easy to use, semi-automatic workflow that combines biomedical CFD results and subsequent supplementary data for immersive viewing and use as a collaborative tool between engineers and clinicians by more easily and rapidly being able to view all available data. The presentation below is similar to prior work advancing methods for CFD related to boundary condition developments (9, 10) in that the utility of the method is presented first in a simple example, and then extended to several specific applications. Application of the workflow is demonstrated across clinical research areas including: the aorta with application to congenital cardiovascular disease, the Circle of Willis with respect to cerebral aneurysms, and the nasal airway for surgical treatment planning. The resulting workflow also aimed to permit viewing across platforms spanning a range of cost and development considerations including a large-scale CAVE-type (CAVE Automatic Virtual Environment) (11, 12) (∼$1.5 million clustered approach), stereoscopic projector (∼$5,000 single processor approach) and HMD (∼$500 mobile approach).

## 2. Materials and methods

The methods below describe steps in the workflow for use with hardware capable of generating and interacting with objects within a virtual environment. All data featured were obtained following Institutional Animal Care and Use Committee (IACUC) approval from the Medical College of Wisconsin and Marquette University (Application 1), and Institutional Review Board (IRB) approval from the University of California San Francisco (Application 2) or the Medical College of Wisconsin (Application 3).

### 2.1. Platforms for immersive viewing

Viewing CFD results immersively requires access to specific hardware. Common types of devices used in the current work included several HMD, a large-scale IVE, and standalone stereoscopic projectors. A HMD includes equipment that is worn by a user to display content directly in front of the eyes. The first HMD was developed in 1968 and subsequent upgrades continued over the next five decades. However, in many cases the devices either lacked functionality, quality and or were too costly (13). Beginning in the 2010's, companies like Samsung and Oculus (now Meta Quest) helped revitalize interest in immersive visualization and VR with HMD product lines that were less expensive, more accessible and provided a higher quality that had been possible previously. All data analysis is precomputed for the current workflow, which enables the visualization to run on low end HMDs such as the Gear VR or Oculus Go, as well as the Oculus Rift on moderately powerful desktop PCs.

Large-scale IVEs can offer advantages over HMDs. For example, large-scale IVEs often facilitate simultaneous experiences with other end users. Large-scale IVEs range in shape and size, each designed for unique purposes (14). Examples include projection-based cylindrical or dome structures, 4–6 walled CAVE-type systems and panel-based system with narrow bezels (15). Wearing system-specific glasses within such IVEs allows for stereoscopic viewing of content. One example is the MARquette Visualization Laboratory (MARVL) within the Opus College of Engineering at Marquette University (16). MARVL is a $1.2 million, 1,700 ft^2^ facility with hardware and software that aims to create a sense of presence within content through stereoscopic viewing for improved depth cues, surround sound, and motion tracking. MARVL has space for over 30 people to view content that has been filmed, reconstructed or created computationally.

Standalone stereoscopic projectors can also serve as virtual environments. This hardware offers a less expensive alternative to large-scale IVEs, while still offering stereoscopic and collaborative viewing (17). Depending on the installation, standalone stereoscopic projectors may not generate as large of a projection as multi-display systems so a user looking directly at the screen from some distance may naturally conclude they are in the real world (18) based on cues noted in his or her peripheral vision.

### 2.2. Workflow requirements

**Figure 1:**
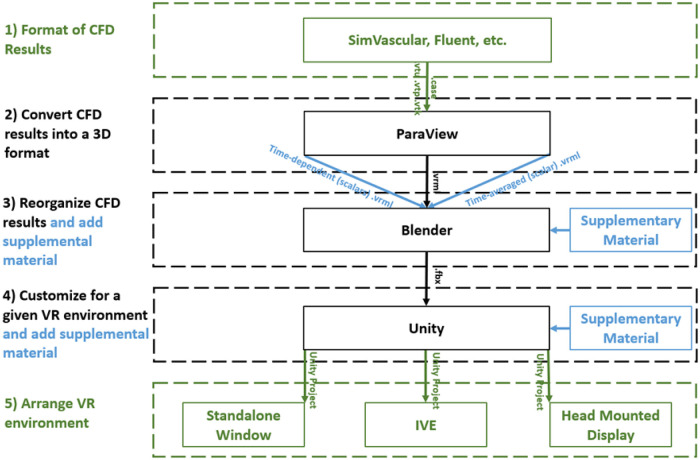
Diagram of the immersive visualization workflow after a CFD simulation is complete. CFD results in a (1) format of the simulation software are (2) converted into .vrml files (type of 3D geometry) before being (3) reorganized in Blender where supplementary material can be added. Simulation results and supplementary material are (4) customized within Unity for the given application and then (5) arranged for a given immersive virtual environment. black = necessary steps, blue = optional steps, green = user selects one of the options.

The workflow described here to display and interact with CFD simulation results within immersive environments has five steps (Figure 1): (1) format, (2) convert, (3) reorganize, (4) customize, and (5) arrange. The workflow is compatible with a variety of CFD software packages and uses open source packages once the CFD results are obtained. For a standard multi-time step CFD simulation resulting in scalars such as pressure, time-average wall shear stress (TAWSS), oscillatory shear index (OSI) as well as animated vector information in the form of streamlines or glyphs (i.e., 3D arrows originating from data points that are colored based on scalar values), the workflow is almost fully automated with the user only needing to complete small tasks and run scripts for each step.

Requirements for the workflow presented above were determined based on the simulation and visualization needs of investigators within the Department of Biomedical Engineering at Marquette University/Medical College of Wisconsin and collaborating clinical divisions, while keeping potential derivative projects in mind. Based on programs and input from associated researchers, the workflow was designed to facilitate viewing of CFD results using a diverse set of hardware including HMDs, IVEs and standalone stereoscopic projectors. For automation purposes, the main Unity template used in part 5 of the workflow is geared to work on systems using the Oculus software development kit (SDK). SDK are libraries of code and examples that help aid the developers in building an application for a specific HMD or IVE. For this workflow, modest alterations can be made to the template to address variances in a platform's SDK if a particular CFD simulation is to be viewed using an IVE or other type of HMD such as the Microsoft HoloLens. To date, the workflow has been successfully tested on VR-based HMD's (e.g., Samsung Gear VR and Oculus Rift), the AR-based Microsoft HoloLens, as well as a large-scale IVE and standalone stereoscopic projector within MARVL.

### 2.3. Format of CFD results

The workflow was designed to accommodate common CFD software packages such as the commercial software Fluent (ANSYS Inc; Canonsburg, PA) and open source software SimVascular (simtk.org). These software packages yield results that can be saved in formats such as .vtk, .vtu, .vtm or .cas files. Given the potential interchangeability and capability of geometry, mesh and simulation results files, it is possible the workflow could also work for other software packages that can be saved, or converted into, one of these formats. The aforementioned files facilitate viewing CFD results on a 2D display but are not natively compatible with IVEs. The steps within the workflow are designed to use supporting software packages that are common within academic, engineering and digital design facilities. Table 1 lists the supporting packages that are required by the workflow, along with the function being performed by each package.

**Table 1:** Programs used in the current workflow and their function in immersively viewing CFD results.

	Function
ParaView	Convert CFD results into a 3D format compatible with immersive visualization
Blender	Reorganize 3D format files and add supplementary data
Unity	Animation, custom interaction, add supplementary data and application used to view CFD results immersively

As an initial test case, we present methods associated with the workflow based on a CFD simulation run using a cylinder phantom. These data are from a microfocal x-ray CT scan of PE-240 tubing as discussed in detail elsewhere (19). For the current work, the imaging data was segmented, lofted into a 3D model, meshed, and an associated simulation was run using SimVascular. The pulsatile inflow waveform used for the simulation was from prior literature of a similarly sized vessel (20). The walls were considered rigid and a 3-element-Windkessel model was applied to the outlet thereby allowing pressure within the cylinder to range between the normal diastolic and systolic values of 80 and 120 mmHg typically measured in a healthy adult. Simulation results were output as a series of 20 .vtu files. The following figures in the methods section were generated from these results for the initial test case.

### 2.4. Convert CFD results into a 3D format

The majority of CFD simulations display flow patterns over some duration of physical time that often represents one cycle of the event being studied (e.g., one heartbeat or one breathing cycle). Velocity results are commonly shown using streamlines or glyphs. To view the streamlines or glyphs in an immersive environment, the CFD results need to be converted into a form that stores 3D geometry, vertex magnitude, and is compatible with the selected 3D gaming engine. The number of glyphs or streamlines to be implemented is a balance between end user preference of aesthetics and ensuring the visualization is not so complex that it is no longer performant enough for the associated hardware. Some guidance on specific details implemented previously can be found elsewhere (6). For the workflow's purpose, streamlines and glyphs for each time step are converted into .vrml files using ParaView version 5.4.2 (Kitware, Inc; Clifton Park, NY). ParaView is an open source software package for scientific and interactive visualization. It was chosen for its ease of use in monoscopically visualizing CFD results before their conversion and because it also has excellent utility as a file conversion tool. ParaView does support stereoscopic viewing, but with several limitations. For example, it may require a special build on some IVE's (16), and may not be available natively with some HMDs to date. Moreover, there is often a lag when viewing consecutive time steps and the viewer is unable to easily view supplementary data sources. The current workflow uses a custom Python script, within ParaView (version 5.4.2), to save each time step as a .vrml file.

### 2.5. Reorganize CFD results

**Figure 2:**
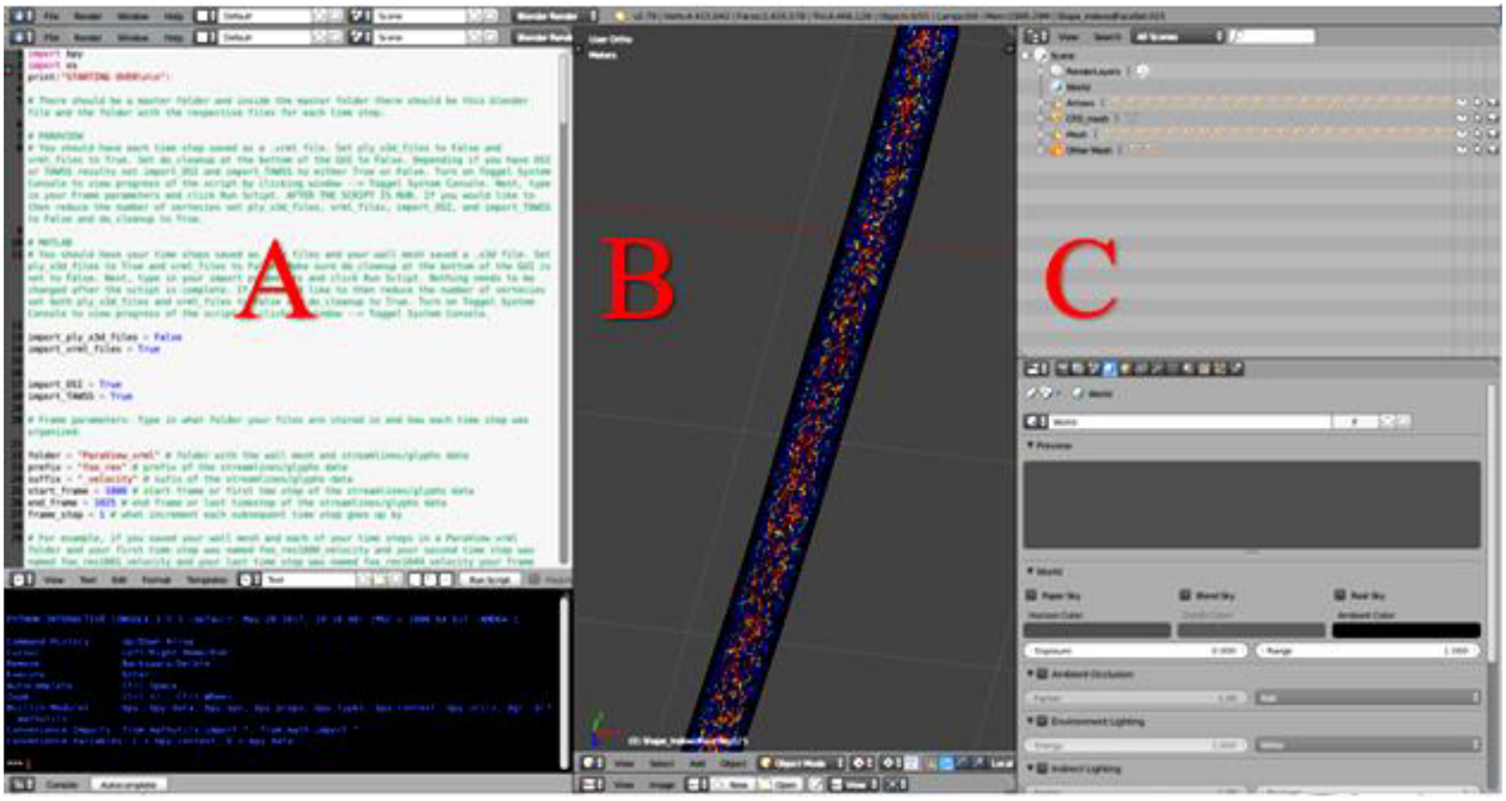
Screenshot of Blender's interface depicting CFD simulation details from a cylinder phantom. (**A**) Blender's Text Editor where a Python script is used to reorganize the simulation details and reduce the number of vertices. (**B**) Blender's 3D View which displays every object of the simulation. Objects will be turned on or off within Unity as part of a later step, and (**C**) Blender's Outliner of parent-child relationships where each object may be edited.

Blender, (Blender Foundation; Amsterdam, Netherlands) is an open-source graphics program used to rectify the import and export formats between ParaView and Unity. ParaView's exportable 3D formats are .x3d or .vrml. In contrast, Unity's suitable importable 3D formats are .fbx, dao, .3ds, .dxf, .obj and .skp. For the current workflow, Blender takes .vrml files exported from ParaView and ultimately exports the entire CFD simulation as a .fbx file for import into Unity. Within Blender all model objects are set to have a consistent scale and default orientation, and their origin is established in a sensible position near an object's center of gravity. All the objects and vertex colors ultimately used during an animation for immersive viewing are created in one Blender project (Figure 2).

When preparing CFD simulations that have multiple time steps, it is important for the workflow that each time step is uploaded in the correct 3D space. To ensure proper object positioning, a Python script was written for implementation in Blender's text editor. The script re-creates the original CFD simulation, sequencing the 3D-geometry-time-steps inside the wall mesh and creates proper parent-child relationships that are necessary when the Blender project is loaded into Unity. At the top of the Python script, shown in Figure 2A, is a set of instructions. In addition to re-creating and organizing the simulation, the Python script also creates a wire-frame wall-mesh game object, UV maps the wall mesh (technique used to wrap a 2D texture on a 3D model), and deletes unnecessary cameras and lamps that are rooted in the .vrml files.

It is common for a CFD model's mesh to have duplicate structures (i.e., a double-sided mesh). When viewing CFD results immersively, an excessive number of vertices can slow down the frame rate of the Unity project, which can contribute to simulator sickness (21, 22) [i.e., motion sickness thought to result when sensory cues receive conflicting inputs from visual vs. vestibular systems (23, 24)]. If the wall mesh is visible in the 3D view without glyphs or streamlines, this indicates a secondary script in the workflow should be run to remove duplicate structures and consequently reduce the number of vertices. The secondary script may also be run regardless of whether the wall mesh is double-sided if the user would like to reduce the number of vertices. Lastly, the Blender file is exported as an .fbx file and loaded into Unity.

The ability to add related supplementary data sources to the viewing of CFD simulation results is one of the main reasons why our CFD researchers and collaborators created the current workflow for immersive visualization. Importantly, supplementary data such as medical imaging can provide valuable information on surrounding structures and tissues, thus combining anatomical and functional results for comprehensive analysis. Depending on the type of data the user wishes to display as supplementary material, it can be added in either the Blender project or Unity template. For example, in the current workflow and template, volumetric imaging data (i.e., CT, MRI) used to create a CFD model and consisting of a stack of slices are imported in Blender while a flow waveform created in a program such as Excel (Microsoft; Redmond, Washington) is added later in Unity.

### 2.6. Customize for the application

The Unity game engine is used in the workflow for its speed, flexibility and ability to integrate data from multiple sources. The combination of Blender and Unity have become the basis for all projects conducted in MARVL to date (16). After the models are prepared in Blender, it is a straightforward procedure to import them into Unity. An environment is created to house models, the models are positioned in the scene, lighting is established, and supplementary features or data are added as needed for a particular application.

For the current workflow, a pre-programed automated Unity template was created for viewing of CFD results immersively using a standalone stereoscopic projector (e.g., Gear VR or Oculus Rift). If the user prefers to view their content using a different HMD or a large-scale IVE, changes to the Unity scene, scripts, and/or custom packages are required. As seen in Table 2, the prefabricated automated Unity template has minimum and optional outputs with additional functionality. The Unity template, *via* automated code, animates the streamlines/glyphs, animates/changes the wall mesh texture for scalar quantities (i.e., pressure, TAWSS, OSI), and toggles through volumetric imaging data. The template also includes a basic environmental audio system containing prerecorded ultrasound samples from several locations within the blood flow domain over a cardiac cycle that have been synchronized to the expected animation. Importing and animating a flow waveform and assigning proper scales (e.g., ranges and units) to the legends for indices being visualized are not automated. However, the approach is straightforward and can be accomplished quickly while not requiring alteration to any code. If the user does not have a full set of supplementary data, the Unity template still works. The initial script to set up the CFD visualization, called an editor script, recognizes how little or how much information is rooted in the .fbx file created in Blender.

**Table 2:** Minimum and optional output, along with additional functionality, of the unity template. Italicized text indicates the user must manually alter the data into the unity prefabricated template.

Minimum Output	Optional Output	Additional Functionality
Animated streamlines or glyphs with *legends*	Toggle between surface (i.e. wall) scalars including pressure, TAWSS, and OSI	*View complementary data such as medical imaging data, data patient, and local structural (e.g. histology) or functional (i.e. myography)*
Fly throughout simulation results	*Animate inlet boundary condition waveform*	*Toggle view between multiple cases or instances (e.g. pre/post treatment)*
Transparent map of current location within the immersive space	Toggle through volumetric imaging data	*Fly down set track*

**Figure 3:**
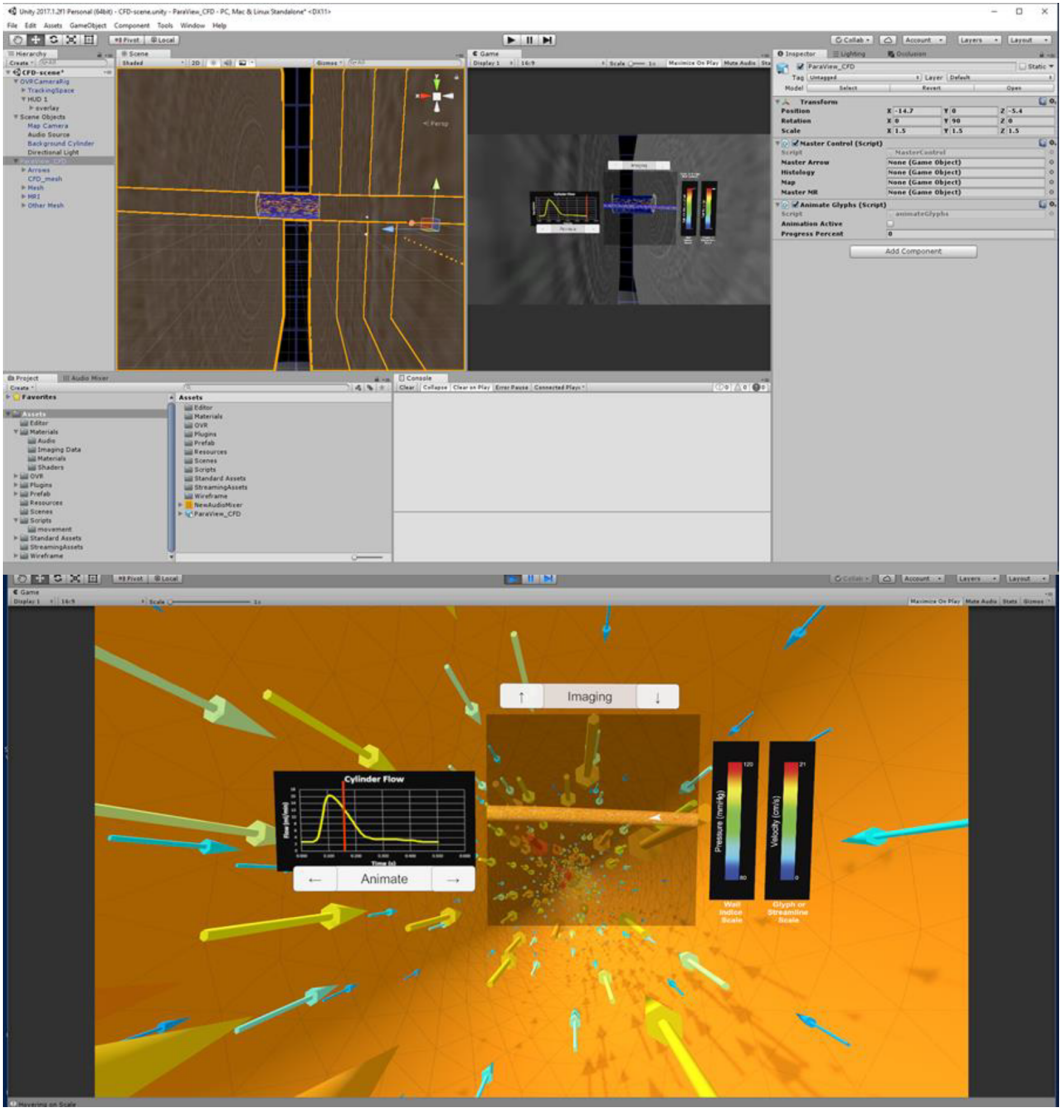
Screenshots of Unity's interface upon completing the steps described for the cylinder CFD simulation. “Play mode” is shown in the top panel with a standalone stereoscopic projector output shown in the bottom panel.

Three tasks are required to complete the automation portion of the Unity template: (1) import the .fbx file created in Blender, (2) scale, rotate, and reposition the simulation assets to a convenient location, and (3) run the pre-written editor script that recognizes the type of information in the .fbx file. This script then tags and assigns game objects. Once a user manually sets up data such as the flow waveform and legends for each index, the Unity scene should appear similar to Figure 3 (top), which displays assets consistent with the maximum output listed in Table 2. Figure 3 (bottom) is a screenshot of the Unity scene when data in Figure 3 (top) is in “Play mode”.

### 2.7. Arrange for a given IVE

When in the virtual environment, interaction is achieved *via* selected buttons included in the Unity scene. For example, if the user includes the optional output and function for the Unity template (Table 2), the clickable buttons and scales are displayed as in Figure 3B. In the absence of volumetric imaging data within the .fbx file, for example, the imaging up and down arrow buttons would not appear in the Unity scene after the editor script was run.

**Figure 4:**
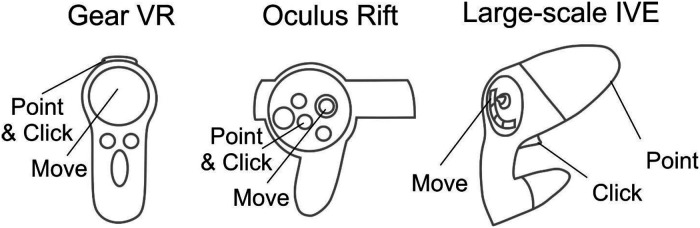
Controls implemented in the workflow. Controls for the Gear VR (left), Oculus Rift (middle), and large-scale immersive virtual environment *via* FlyStick 2 (right) are shown. The trackable controllers are viewed as an orange beam in the Unity Scene. The user positions the controller in space to point the beam at a portion of the results to be viewed and then make a selection *via* the buttons shown.

Different HMDs use their corresponding interaction devices to foster movement and selection (Figure 4). For the Gear VR and Oculus Rift, the trackable controller is viewed as an orange wand virtually in the visualization. When the user positions the wand over one of the buttons, it turns yellow to indicate an ability to be selected. When the user selects the button, the corresponding action is applied to the simulation. The current workflow has designated the gaming pad to conduct movement for the Gear VR, while the Oculus Rift uses the right analog stick for movement. Steering is achieved by the user's head rotation for both the Gear VR and Oculus Rift. For interaction, movement and steering within MARVL's large-scale IVE, the Unity template was altered to use the FlyStick2 wireless interaction device (Advanced Realtime Tracking; Weilheim, Germany). The current template uses standard mouse and keyboard controls when viewing CFD results using a standalone stereoscopic projector.

## 3. Results

The workflow discussed above was tested with three different clinical case studies from CFD researchers at our institution who had an interest in expanding viewing of CFD results in immersive environments with complementary data. The case studies aim to demonstrate its potential utility of the workflow across clinical research areas and using virtual environments spanning a range of cost and development considerations (Table 3). As discussed in more detail below, these include hemodynamics imparted on the thoracic aorta with respect to a congenital cardiovascular disease, cerebral vasculature with application to aneurysm progression, and nasal airflow with application to virtual surgery planning. All data featured in the current work was accessed following applicable Institutional Animal Care and Use Committee or Institutional Review Board approvals.

**Table 3:** Portions of workflow implemented in the three featured CFD application examples.

	Coarctation of the Aorta	Cerebral Aneurysm	Nasal Airway
*CFD Software*	SimVascular	Fluent	Fluent
*Velocity Visualization*	Glyphs	Streamlines	Streamlines
*Unity template*	Optional (CFD results, legends, inflow waveform, imaging data) plus additions for group/treatment viewing	Minimum (CFD results and legend)	Minimum (CFD results and legend) plus additions for pre/post treatment viewing

The thoracic aorta simulation featured here leverages data, methods and results discussed in detail elsewhere (25, 26, 27, 28). Briefly, the simulation focuses on coarctation of the aorta (CoA), one of the most common congenital cardiovascular defects (29). It most often presents as a narrowing just downstream of the left subclavian artery. CoA is associated with decreased life expectancy, and hypertension presents in most patients despite treatment (30). Mechanically induced structural and functional vascular changes are implicated in CoA, but the mechanisms by which stimuli lead to vascular changes such as altered smooth muscle phenotypic expression and endothelial dysfunction ultimately resulting in hypertension are not fully understood. Using a clinically representative rabbit model of the untreated (i.e., CoA) and treated (i.e., Corrected) conditions as compared to Controls, simulation results provide detailed mechanical stimuli and vascular alterations from a 20 mmHg blood pressure (BP) gradient, which is the current putative treatment threshold implemented clinically. Available data specifically includes blood flow velocity, pressure, TAWSS, OSI, magnetic resonance angiography (MRA), and blood flow waveforms from associated CFD simulations. The impact of mechanical stimuli on the expression of key proteins impacting vascular remodeling, relaxation, and stiffness is also available for locations above and below the coarctation in each group. This includes Verhoeff-Van Gieson (VVG) and immunohistochemical (IHC) images, as well as myography data of vascular function in response to contractile or relaxation agents (27). Our objective with these data as a case study in the current work was to immersively and simultaneously compare CFD and experimental results. Examples from rabbits in each group (i.e., CoA, Corrected, and Control) used the same methods outlined above to create versions of the results for immersive viewing within available virtual environments.

A second clinical application area in the current work features data from the Circle of Willis, which is a common area of cerebral aneurysm formation. Local hemodynamic forces play an important role in aneurysm growth and rupture (31); therefore, obtaining patient-specific flow metrics can help in diagnostics and treatment planning. In this case, flow simulations were conducted for an aneurysm of the basilar artery. In addition to a pulsatile CFD simulation, available data include time-resolved, three-directional phase-contrast MRI (4D flow MRI) velocity measurements (32, 33). While both CFD and 4D flow MRI provide valuable information on complex flow patterns in aneurysms, both approaches have limitations and advantages. The accuracy of 4D flow MRI is affected by limited spatiotemporal resolution, dynamic range of velocities, and image noise. CFD modeling, while providing superior accuracy, relies on modeling assumptions, e.g., laminar or turbulent flow regime, blood properties, and boundary conditions that may be not physiologically representative. Hence, there is potential utility in interacting with detailed spatiotemporal data to compare CFD simulation and *in vivo* 4D flow MRI measurements for the same aneurysm using an immersive approach. The end goal of these data as a case study in the current work was to view the cerebral aneurysm in its natural anatomical orientation and observe the complex intra-aneurysmal flow patterns obtained from the imaging and modeling modalities.

A third case study below features CFD simulations of nasal airflow designed to effectively communicate virtual surgery modeling predictions and help surgeons determine which approach may be optimal for patients with nasal airway obstruction (34). Two CFD simulations were used. The first simulation shows the nasal airflow patterns in a patient with nasal obstruction due to deviated septum (pre-surgery). The second simulation was from the same patient, but after virtual surgery to correct the obstruction (i.e., post-surgery). Both CFD simulations included pressure and velocity information from steady simulations appropriate for this portion of the anatomy. The end goal of this data was to immersively travel through the nasal cavity while being able to compare the pre and post-surgery states instantaneously. A representative surface model from a scan of a human head and *x*, *y*, and *z* coordinates that would be later used to create a set animation track were also available from collaborating researchers.

### 3.1. Application 1 - arterial hemodynamics with application to coarctation of the aorta

#### 3.1.1. Format of CFD results

MRA imaging data from the thoracic aorta of representative rabbits in each group (Control, CoA, and Corrected) were obtained using GE Healthcare's 3-T Sigma Excite MRI scanner at the Medical College of Wisconsin. Computational models were created from this data using SimVascular as discussed elsewhere (27, 28). Corresponding CFD results from each simulation were represented as twenty-five .vtu files representing instantaneous results at regular intervals spanning the duration of a single cardiac cycle. Two .vtk files were also created from this instantaneous data, one each for TAWSS and OSI. Simulation results from representative rabbits in each group all used the same workflow above as discussed in more detail below.

#### 3.1.2. Convert CFD results into 3D format

Collaborating researchers for this case study desired to view instantaneous pressure, along with the scalars of TAWSS and OSI, and velocity results as vector representations. The glyph workflow was therefore used to implement the following details. First, each .vtu file was loaded into ParaView. Next, pressure was assigned to display on the aortic wall for each time step. Glyphs were then added using ParaView's glyph tool at each time step. The Python script was run, during which each .vtu file was saved as a .vrml file containing velocity (glyphs) and pressure (wall) information, respectively. OSI and TAWSS .vtk files were then separately loaded into ParaView and exported as .vrml files.

#### 3.1.3. Reorganize CFD results and add supplementary data

The provided Blender template was opened, instructions were completed, and the script was run. Each time step took approximately fifteen seconds to load.

After the CFD simulation was reorganized in Blender, the Blender scene was altered to display supplementary data. First, image slices representing volumetric MRA data were overlapped on the wall mesh. Then, the aorta was conceptually divided into two regions within the immersive space: (1) a proximal region above the spatial location of the coarctation and (2) a region distal to the coarctation location. Within Blender, the scene was then saved and exported as a .fbx file. The inflow waveform was then created in a readily available graphics program (e.g., Microsoft Excel, SigmaPlot, or similar) and saved as a .png image. A .png image was also created that included a montage representation of the VVG, IHC, and myography data taken from the proximal and distal regions of the aorta. Finally, each MRA imaging slice, flow waveform PNG image, regional PNG images, and .fbx file were then copied into the Unity template.

#### 3.1.4. Customize for CoA data

The Unity template refreshed upon opening, which allowed each PNG image and the .fbx file (now a prefab) to appear. Next, the CFD simulation prefab was dropped into the hierarchy where it was properly scaled and repositioned. The editor script to set up the scene was run to properly tag glyphs, the wall mesh, and volumetric image slices. The flow waveform and legends for each indices (i.e., pressure, TAWSS, OSI) of interest were then set up.

#### 3.1.5. Arrange for a given IVE

**Figure 5:**
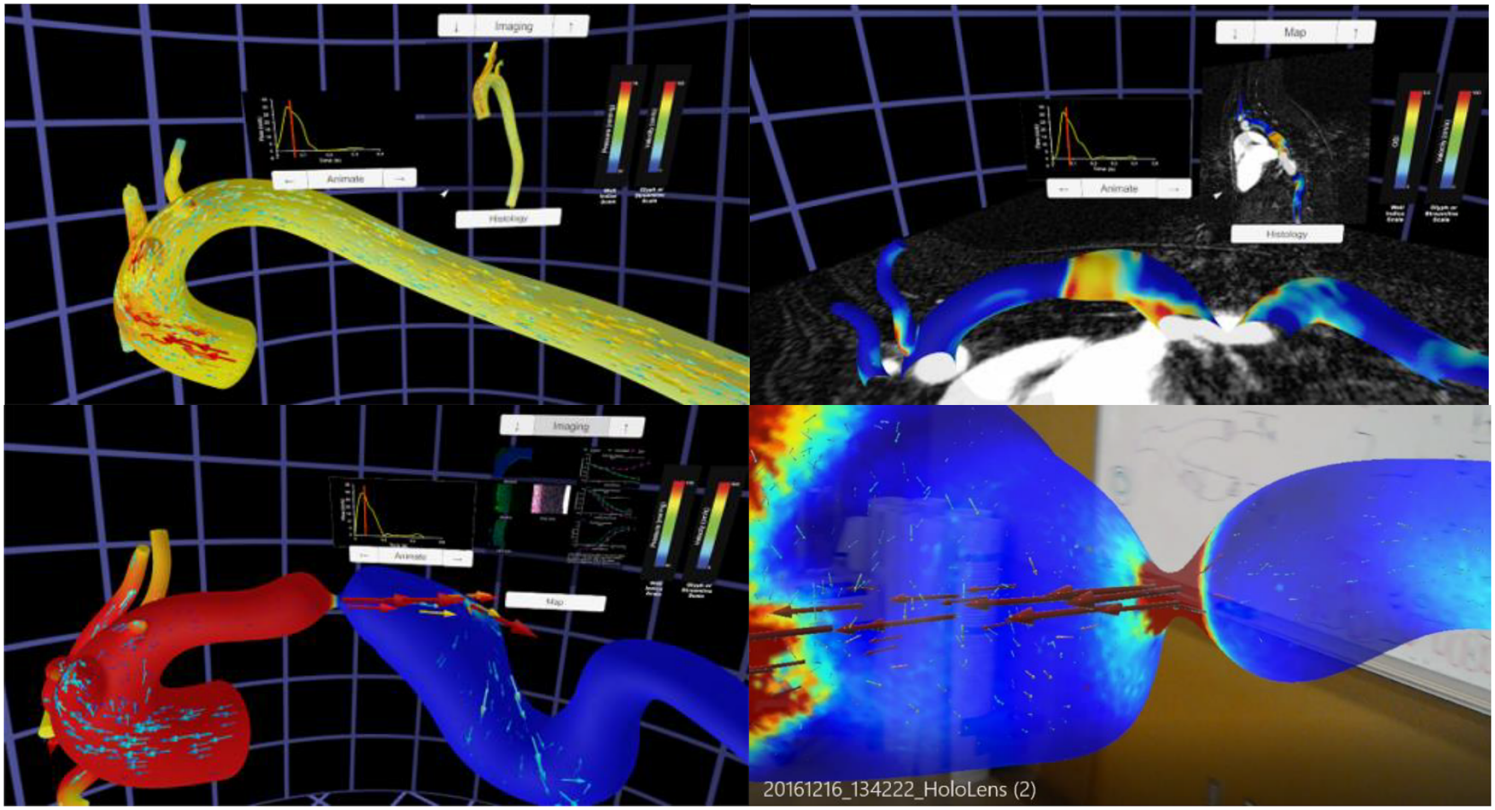
Screenshots from immersive viewing of a thoracic aorta data with application to coarctation of the aorta (CoA). Results shown using the Oculus Rift include those from a user simultaneously viewing instantaneous velocity vectors and pressure from a simulation in the Control group (top left), peak systolic pressure and velocity from a simulation in the CoA group (bottom left) together with local immunohistochemistry (i.e., structure) and myography (i.e., function) data, and oscillatory shear index and corresponding imaging data from a simulation of the Corrected group (top right). All results have a marker on associated inflow waveforms that indicate current time during the cardiac cycle. The current viewing location within the geometry and legends for each index are also present in each panel shown. The bottom right image shows a HoloLens after processing data from this application through the workflow and viewing in augmented reality within a research laboratory setting.

The Unity template was slightly altered to display the montage of structure and function data from locations above and below the coarctation. The alterations allowed these montages to switch if a user was originally in the proximal end of the CoA and then flew through the CoA into the distal end, or vice versa. These data have also been subsequently viewed immersively on the Gear VR, Microsoft HoloLens and large-scale IVE within MARVL. The final versions of each thoracic aortic simulation using the Oculus Rift and HoloLens are shown in Figure 5.

### 3.2. Application 2 - cerebral vasculature hemodynamics for aneurysm progression

#### 3.2.1. Format of CFD results

This case study utilized contrast-enhanced MRA and three-directional phase-contrast MR velocimetry (4D flow MRI) datasets obtained for a basilar tip aneurysm patient at the University of California San Francisco. Patient-specific vascular geometries were obtained from the MRA data using in-house segmentation tools. The region of interest was then defined, noise was reduced, and the surfaces were converted into IGES format using a 3D modeling software Geomagic Design (3D systems; Rock Hill, South Carolina). The computational mesh on the domain was created using Hypermesh (Altair; Troy, Michigan) and the transient, incompressible Navier-Stokes equations were solved using a finite volume solver Fluent (ANSYS Inc; Canonsburg, PA) (32). The inflow and outflow boundary conditions were obtained from the 4D flow MRI data and used to prescribe time-dependent waveforms for the CFD solver. The simulation data were then exported into VTK format for data visualization. *In vivo* 4D flow MRI data containing velocity components through the cardiac cycle were processed using ParaView and EnSight (ANSYS Inc; Canonsburg, PA) (33). Both the CFD simulation and 4D flow MRI data were presented to MARVL as .cas files from Fluent, only including velocity information. The CFD simulation had sixty-six files representing instantaneous results at regular intervals spanning the duration of a single cardiac cycle and the 4D flow MRI simulation had twenty files over the same duration.

#### 3.2.2. Convert CFD results into 3D format

The streamline workflow was used for this case study at the request of the collaborator. First, the CFD .cas file was loaded into ParaView. Tube-based streamlines were then added at each file using ParaView's stream tracer tool and tube filter. Finally, each of the sixty-six files in the cardiac cycle were saved as .vrml files using the custom Python script. The same process was performed for the 4D flow MRI .cas file, where each of the twenty files representing the cardiac cycle were saved as .vrml files. The corresponding geometry data was not extracted from the 4D flow MRI data due to the limited spatial resolution of the images. Therefore, the wall mesh from CFD data was manually overlaid on the MRI velocity data in Unity to provide a frame of reference for the 4D flow data.

#### 3.2.3. Reorganize CFD results

The following steps were implemented with data from CFD and 4D flow MRI. First, the Blender template was opened, and the associated script was run. Each time step took approximately twelve seconds to load. After the CFD simulation was created, it was noted that its wall mesh was double-sided, and each time step had a large number of vertices. Therefore, the secondary Blender Python script was run to remove duplicate mesh structures and decrease the vertex count. After the 4D flow data was processed, slight alterations were made to the Blender scene to account for the lack of an associated all wall mesh, which was later added in Unity. Finally, the Blender project was exported as a .fbx file.

#### 3.2.4. Customize for cerebral aneurysm data

Collaborating researchers did not request viewing of any supplementary data with the CFD and 4D flow MRI results. The results were therefore prepared for the immersive environment by opening and refreshing the Unity template, causing the .fbx file (now a prefab) to appear. Next, the prefab was added into the hierarchy where it was properly scaled and repositioned within the virtual world so that its presentation was at a desired scale and elements such as the background, cameras, and user interface were located in reasonable positions for the experience. The wall mesh was originally solid white, which was thought to be suboptimal. Instead, a red material was created and applied to the wall mesh to better represent its classic association with arteries. To add the CFD wall mesh to the 4D flow data, the wall mesh of the CFD simulation was manually overlaid on the 4D flow MRI data. Next, the editor script was run allowing the wall mesh and streamlines to be tagged, and scripts were assigned to the various game objects. Finally, the velocity legend was labeled using scales consistent across the types of data.

#### 3.2.5. Arrange for a given IVE

**Figure 6:**
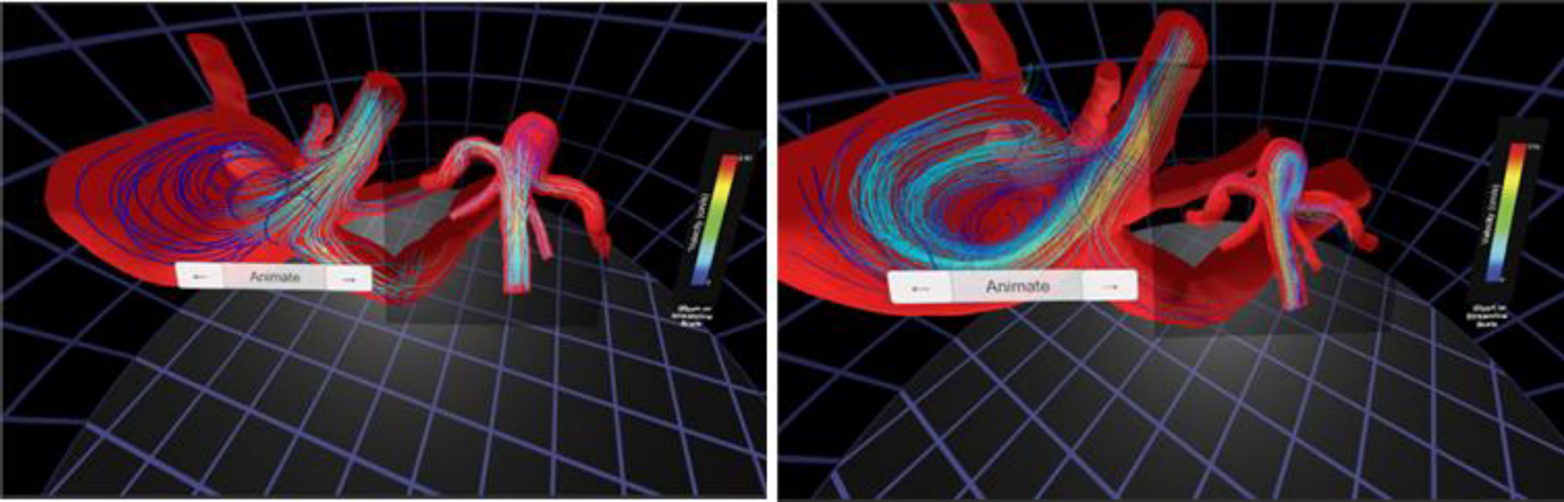
Screenshots from immersive viewing of cerebral vascular data with application to characterization of aneurysmal hemodynamics using the Oculus Rift. Streamlines of velocity from CFD simulation results (left) are shown alongside those from 4D flow (right). The current viewing location within the geometry and legends for velocity are also present in each panel shown.

Standard interaction was sufficient for this case study. The resulting versions of the CFD simulation and 4D flow data visualized using the Oculus Rift are displayed in Figure 6. These data have also subsequently been viewed immersively in MARVL's large-scale IVE.

### 3.3. Application 3 - nasal airflow for virtual treatment planning

#### 3.3.1. Format of CFD results

The two nasal airflow simulations originated from the same obstructed nasal cavity CT scan obtained at the Medical College of Wisconsin. Using Mimics (Materialise; Leuven, Belgium), CT data was segmented to create a 3D model representing the nasal passage prior to surgery (i.e., pre-surgery). A virtual septoplasty (i.e., post-surgery) model was also created in Mimics to represent the surgical outcome after the septal deviation was corrected. Both 3D models were then meshed using ICEM-CFD (ANSYS Inc; Canonsburg, PA) and steady CFD simulations were conducted using Fluent. Resulting CFD simulation results were available as separate .cas files for each simulation. To foster the collaborators vision of comparatively viewing pre and post-operative results while traveling through the airway, an .stl file was provided that represented a rendered surface model from a scan of a representative human head. Additional steps were required with the current workflow to further achieve the collaborator's vision for immersive visualization of results. These additional steps are discussed in more detail below.

#### 3.3.2. Convert CFD results into 3D format

The workflow was used with the streamlines option to visualize changes in velocity and pressure from the pre- to virtual post-surgery states. First, the respective .cas files (simulations) and .stl files (anatomy) were loaded into ParaView. A set of tubed-streamlines were applied to the pre-surgery and post-surgery data using ParaView's stream tracer tool and tube filter. Both indexes were selected so clinicians could switch between velocity and pressure information as discussed below. Seven .vrml files were exported representing: (1) anatomical wall mesh of a head, (2) pre-surgery and (3) post-surgery nasal cavity mesh, (4) pre-surgery and (5) post-surgery streamlines colored by velocity magnitude, and (6) pre-surgery and (7) post-surgery streamlines colored by pressure magnitude.

#### 3.3.3. Reorganize CFD results

As with the prior case studies discussed above, the Blender template was opened, and the associated scripts were run. Each .vrml file took about fifteen seconds to load. The wall mesh for both the pre- and post-surgery models included additional geometry at the top of the nasal cavity that was unnecessary for the collaborators' viewing objectives. This unnecessary geometry was therefore manually deleted using Blender.

#### 3.3.4. Customize for nasal airflow data

Supplementary data for this case comprised of *x*, *y*, and *z* coordinates that clinical collaborators desired to use as a specific track for a camera within the immersive space to move along (i.e., virtual endoscope). The coordinates were provided as two .txt files, corresponding to the right and left nasal cavities. After the project was reorganized in Blender, the scene was altered to add coordinates for vertex points along each track using Blender's text editor. The vertices were parented to two cameras, one for the left nasal cavity and one for the right nasal cavity. The Blender scene was then exported as an .fbx file. The Unity template was then opened and refreshed, causing the .fbx file (now considered a prefab) to appear in the Unity template. Next, the prefab was added into the hierarchy where it was properly scaled and repositioned.

#### 3.3.5. Arrange for a given IVE

**Figure 7:**
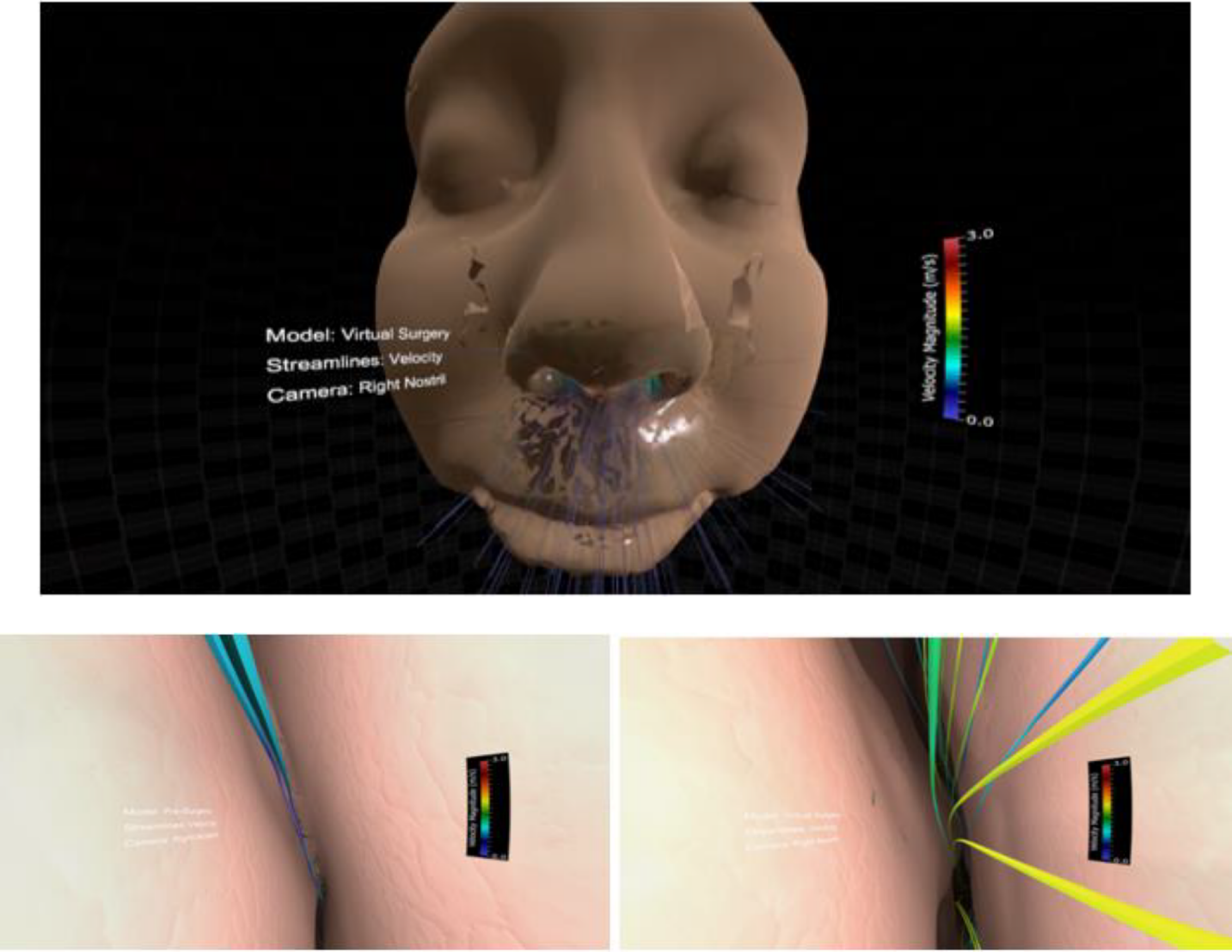
Screenshots from immersive viewing of nasal airway data with application to virtual treatment planning using the Oculus Rift. Initial view of the scene using a representative geometry (top) along with views of velocity streamlines from inside the right nasal cavity pre-surgery (lower left) and post-surgery (lower right). Note the increase in velocity post-surgery following removal of the obstructed region resulting in increased regional flow.

This project was slightly different from the two reviewed above and therefore required modest alteration. First, the Unity camera head node was parented to the Blender camera. Next, scripts were written to (1) toggle between the pre and post-surgery results, (2) toggle between velocity and pressure streamlines, and (3) start and switch the camera animation created in Blender. Finally, legends were added to optimize on the range of values for pressure and velocity in each simulation. The resulting Unity scene, in play mode, initially appears as Figure 7. The figure also shows the view inside the left or right nasal cavity when the user toggles between pre and post-surgery CFD simulation results, observing the changes in geometry, velocity, and pressure information using the Oculus Rift.

## 4. Discussion

Researchers using CFD for biomedical applications can spend weeks or months creating realistic geometric models, determining appropriate boundary conditions, running simulations, and post-processing results (3). In most cases, only a fraction of the simulation results are studied in detail, and results are typically viewed on a 2D display, presented at one timepoint from a single viewing angle, or at several timepoints. Currently available software tools allow for viewing of a CFD model with its corresponding imaging data and resulting simulation indices. However, spatiotemporal relationships between hemodynamic indices of interest from CFD and important data concerning structure or function are rarely shown together. This shortcoming in concurrent viewing of data may limit our ability to make associations between mechanical stimuli and biological response for a given pathology. VR and AR may alleviate many of these issues through the use of improved depth cues, custom interactivity, and an immersive approach that can create a sense of presence within the data by a researcher (6). Motivated by recent work using Unity to view CFD results for another discipline (8), we sought to develop a rapid, easy to use, semi-automatic workflow that combines biomedical CFD results and supplementary data to be viewed in a wide range of systems. As outlined above, we present methods associated with the workflow using data from a cylinder phantom imaged *via* microfocal x-ray CT (19). Three case studies with different input and output specifications were then presented for several clinical applications viewed across platforms spanning a range of cost and implementation considerations (e.g., size of full-scale IVE vs. HMD).

Our intention is for the workflow to be used as a collaborative tool between CFD researchers and clinicians by more easily and quickly being able to view available data. Data scientists may also find the tool useful. However, our perspective during development of the current workflow was as CFD researchers who needed a set of tools that were not available at the time. Many CFD researchers are students gaining credentials toward careers in industry or academia. With this in mind, ease of use for the current workflow was confirmed by one graduate and one undergraduate student at our institution. While the graduate student had some prior experience running CFD simulations and using ParaView, neither student was familiar with Blender, Unity or methods implemented to view results immersively in VR or AR. Students were presented with a PNG flow waveform and 20 .vtu files previously generated using SimVascular. Thus, the students were presented with the minimum output along with an animated flow waveform, which is part of the Optional Output portion of Table 2. The immersive visualization workflow was successfully completed by the graduate student in one hour and seven minutes. This completion time included processing such as running the Python script in ParaView, running the Python script in Blender, opening Unity, and reloading the .fbx file into the Unity scene. The undergraduate student was then presented with the same data. After study personnel trained the undergraduate student on how to use ParaView, the workflow was successfully completed in fifty-nine minutes, which also included the processing time for each step. As a frame of reference, a user who was proficient in ParaView, Blender and Unity was able to prepare the same data set for immersive viewing in eighteen minutes, including the processing time for each step. Overall, study personnel were satisfied with this result. While this initial and informal feedback does not represent a rigorous usability study, it does suggest the workflow presented here can be used by others in the field as novice researchers with no prior knowledge of Blender or Unity could not likely have easily viewed their simulation results immersively in this short amount of time previously (i.e., ∼1 h).

The current methods and applications highlighted should be interpreted relative to several potential limitations. The aortic coarctation and cerebral aneurysm applications were implemented in a way that allows the vasculature to disappear as the virtual camera nears the location a vessel. In contrast, our collaborators for the nasal airway for surgical treatment planning application requested a perspective consistent with endoscopy using the proposed methods. This highlights the utility of the tools presented to address the needs of collaborators for a given application. It may be possible to script extraction of flow waveforms from ParaView within regions of interest *a priori*. The user could leverage the Python window (for example) to code steps to set a cut plane and engage a filter to integrate velocity for points within the plane. Results could then be implemented in the current workflow as was done for inflow waveforms. Setting the location of imaging data, planes and complementary data is done in Unity as described above. Hence, the workflow may take additional time to implement with multiple imaging data sets if their co-ordinate systems are not aligned with the CFD geometry. The workflow is currently limited to volumetric imaging data from which CFD models were created and data from those modalities from which data can be viewed on a plane positioned within Unity. Immersive visualization of CFD results can be misleading depending on the users viewing angle, which is an important consideration related to the use and applicability of the current workflow. One approach to mitigating this issue within MARVL discussed in detail elsewhere (16) has been to limit the use of head-tracking to situations where a single user is in the IVE. However, for collaborative settings such as those for which the current workflow was developed, we set a virtual camera at a position and orientation representative of a seated person in the center of the room. The stereo axis of each screen is then aligned to the face normal of each screen thereby allowing the audience to visualize a stereo image on all screens, albeit with more pronounced screen boundaries. The CAVE-type implementation within MARVL uses the VR toolkit MiddleVR to supplement some features missing from Unity for use in the CAVE. Like Unity, MiddleVR is not open-source, but both are available under a generous license for educational use and the future may bring fully open-source alternatives. Although Unity is not open source, the project files and resources that rely on it can be shared under an open-source license.

The goal of the current work was to present and disseminate a new tool for enhanced viewing of CFD results and associated data in an IVE. As has been previously recounted to our team, an IVE offers the potential for more real estate with fewer distractions. The approach employed in describing our methods is similar to prior work advancing methods for CFD related to boundary condition developments (9, 10) in that the utility of the method is presented first in a simple example, and then extended to several specific applications. Future work applying the current advancements will strive to quantitatively assess the ability of the methods presented here provide measurable insights, likely using established measures and relative to current trends and challenges (35, 36). It is also important to note that although the workflow has been tested with additional users from our lab having some familiarity with CFD methods, an extensive usability evaluation using the System Usability Scale (SUS) or similar measure has not been conducted to date. As a result, statistical evaluation of data generated by researchers using the workflow is not available to date. Including such information from collaborating labs would be an extensive undertaking involving the coordination of multiple investigators that is beyond the funding, personnel resources and scope of work for our research at the current time. We acknowledge that as a major limitation of the current work, but are optimistic that such feedback and associated data will be obtained intrinsically following the dissemination of the workflow.

The workflow featured requires some processing of CFD simulation results as discussed before immersive versions of the results can be created for viewing. The user can then interact with the perspectives and orientations of these immersive versions, but the current version of the workflow does not allow for additional operations with the simulation results to be performed in real-time. Such interaction is available in other programs (e.g., ParaView), but to our knowledge these programs do not easily allow for inclusion of complementary data. Interestingly, related work has recently been conducted in this area for visualization of CFD simulation results of intracranial arteriovenous malformations (AVM) (37) in which users can interactively block arteries supplying an AVM in VR. Similar to the current workflow, the AVM study also leverages pre-computed CFD simulation results. The inclusion of real-time manipulation of native simulation results immersively *via* user interaction represents a potential area of development for the current workflow moving forward.

The data format from our simulations was in the form of vertex colors, and the UV maps were generated in order to allow those data to be more easily processed. UV maps were generated by baking vertex colors onto 2D image files, not mapping 2D images onto 3D faces, so the UV unwrapping distortion was avoidable by using a large number of UV patches. Swapping image files seemed to be robust and performant, but there are several ways to achieve multiple color maps. For example, alternative methods such as using custom shaders and multiple vertex colors could also likely work with additional scripting.

There are many file formats and software tools that are used for CFD. The choice of file formats implemented here are consistent with those common to researchers using SimVascular, FLUENT and ParaView. The use of other files formats may allow for the elimination of steps presented to date in future iterations of the workflow, or by those researchers starting from a different file format. It is also worth noting that the specific devices featured in the current work may undergo iterations. Hence, the workflow may need to be revisited on a case-by-case basis as new devices become commercially available. Nonetheless, the workflow was also implemented so that immersive visualization of CFD simulation results could be used as a collaborative tool between engineers and clinicians. For example, study personnel have been in meetings with clinical colleagues who have a firm understanding of principles governing fluid flow and their current putative relationships to pathology. In a specific prior meeting with one of these clinical collaborators, study personnel were asked to show time-varying pressure, followed by velocity and then TAWSS using a conventional visualization software. This took a substantial amount of time as the resulting indices from each time step were loaded into memory and rendered. Depending on the availability, proximities and schedules of collaborators, this may be a barrier to collaboration and translation. Implementation of the approaches described results in hardware memory requirements that are far lower than other visualization methods because our results are included in the visualization of objects instead of computed (called and rendered). This is what allows the current methods to run on mobile platforms effectively. The drawback at present is that this approach is less flexible in general. As compared to traditional approaches, rapid and immersive presentation of CFD and complementary data may facilitate mutual understanding between the engineering and clinical collaborators reviewing a case. To underscore results presented for the aortic coarctation study, all related forms of data were presented concurrently using the workflow presented. MRI slices could be toggled on and the user could move through each plane relative to the subject-specific geometry created and associate hemodynamics indices. Similarly, corresponding structural IHC and myography function data from above as compared to below the coarctation were triggered to present based on the location of the virtual camera, and multiple states analogous to control, preoperative and postoperative were available to the user at runtime. To date, no other tool allows for such holistic visualization of results. Having all these data present through such an analysis tool, we believe, is more likely to result in a better understanding between data types and be hypothesis-generating related to future study of the pathology. Study personnel are therefore optimistic that the current workflow will have distinct clinical utility beyond conventional viewing of CFD results. The following informal feedback was obtained by collaborators for each of the case studies presented.

*This would be a valuable education tool for our patients with CoA, which can present very early in life, but also older kids who come in with hypertension. This tool could help them understand the condition better. The VR simulations are a unique, innovative sharing tool I think would increase patient and family engagement. A great way to visually see surgical changes*.

*I see this as a first step in using flow dynamics to predict which aneurysms are more likely to rupture and therefore need to be surgically treated. First we need to visualize the problem, which can be done using the 3D VR model to objectify what is bad and what is good.*

*The nasal airflow visualization was very impressive. The general feedback from those who experienced it was that this would be a very useful educational tool in this context and many other surgical contexts as a way to see the 3D relationships between structures. The addition of the two simulations on top of each other was a great way to visually see surgical changes. Surgery is a very visual field, and this was very helpful. As one becomes more familiar with the CFD information, having this information to visually see in the model was more impressive to surgeons than just seeing the simulations in 2D.*

## 5. Conclusions

Immersive tools offer one approach to extracting more details from biomedical CFD simulation results. However, it is imperative to decrease and simplify associated processing steps to facilitate more widespread use of immersive CFD viewing. The workflow described herein can be used by novice researchers within ∼1 h, demonstrating the ease of the workflow for an audience who may not be familiar with the specific tools used in VR and AR. The workflow is capable of combining biomedical CFD results from a range of pathologies with supplementary clinical data to be viewed in a wide range of immersive environments including an IVE, HMD, and standalone stereoscopic projector. As of the time of publishing, video tutorials for the steps outlined above are provided on YouTube and the workflow is available for download in GitHub. Exploration of these resources can also mitigate the inherent issue present here of trying to replicate multimodal data visualization through print and text-based media. We anticipate that future work will allow for deformable walls and associated imaging data from fluid-structure interaction simulations and additional indices of interest.

## Data Availability

Details related to the original contributions presented in the study are included in the article. Further inquiries can be directed to the corresponding author/s.
